# An acute intervention experimental study on the effects of green and blue environment exposure combined with tai chi exercise on the emotional health of elderly males

**DOI:** 10.3389/fpsyg.2026.1743865

**Published:** 2026-02-04

**Authors:** Maolin Zhang, Yin Zhang, Zhiyao Zhang, Hongmei Sun

**Affiliations:** 1 Wushu School of Shandong Sport University, Jinan, Shandong Province, China; 2 Graduate School of Shandong Sport University, Jinan, Shandong Province, China; 3 College of Sports and Health, Shandong Sport University, Jinan, Shandong Province, China

**Keywords:** green environment, blue environment, tai chi, elderly males, emotion

## Abstract

**Purpose:**

This study investigated the acute effects of green and blue environment exposure combined with Tai Chi exercise on the short-term emotions of elderly males, and comparatively analyzed the characteristics of acute emotional responses to short-term exposure to different environment types paired with Tai Chi exercise, as well as differences in the maintenance of acute emotional effects following subsequent exposure to different environments after the combined intervention.

**Methods:**

Elderly males from Qinlou Subdistrict, Donggang District, Rizhao City, Shandong Province were recruited and assigned to four groups using the random number table method: conventional environment + Tai Chi (TJQ, *N* = 23), green environment + Tai Chi (GTJQ, *N* = 24), blue environment + Tai Chi (BTJQ, *N* = 23), and balanced green-blue environment + Tai Chi (GBTJQ, *N* = 24). An acute longitudinal dual-intervention model of “short-term green and blue exposure + short-term Tai Chi exercise + short-term continuous green and blue exposure” was implemented. Heart rate variability (HRV), blood pressure, and short-term positive and negative emotion indices were measured at four time points: prior to the experiment, after 20-min environmental viewing, immediately post-exercise, and at heart rate recovery to resting level.

**Results:**

Compared with baseline, the GTJQ, BTJQ, and GBTJQ groups exhibited positive changes in HRV, blood pressure, and emotional indices after 20-min environmental viewing. All of the groups (TJQ, GTJQ, BTJQ, and GBTJQ) demonstrated positive changes in these parameters immediately after acute exercise and at heart rate recovery to resting level. The magnitude of acute positive emotional changes across all of the time points followed the order: BTJQ > GBTJQ > GTJQ > TJQ, with the BTJQ group showing the most pronounced changes.

**Conclusion:**

Acute exposure to different types of green and blue environments exerts positive effects on the short-term emotional health of elderly males. On this basis, acute exposure to different green and blue environments in combination with Tai Chi exercise further enhances short-term emotional outcomes in elderly males. Further, following this combined acute effect, continuous exposure to different green and blue environments maintains positive emotional changes for a longer duration during the recovery period. The environment types, ranked by the magnitude of acute positive changes and maintenance effects, are as follows: blue environment > balanced green-blue environment > green environment > conventional environment.

## Introduction

According to the World Population Prospects 2024 report by the United Nations Department of Economic and Social Affairs (United Nations Department of Economic and Social Affairs, 2024), older adults aged 60 and over currently constitute 13% of the global population (approximately 1 billion), a proportion projected to reach 22% (2.1 billion) by 2050. Data from China’s National Bureau of Statistics as of 2022 (National Bureau of Statistics of China, 2021) indicate that adults aged 60 and over in China account for 19.8% of the total population (approximately 280 million), with projections suggesting that this ratio will exceed 30% (over 400 million) by 2035 and peak at approximately 524 million by 2052, marking China’s entry into a severely aging society. The promotion of physical and mental health in older adults has therefore emerged as a critical public health issue worldwide. Aging is accompanied by organ functional decline, psychoneural system aging, and various psychosocial stressors (such as loneliness and economic pressure), which exacerbate mental health problems in older adults (He et al., 2020; Liu et al., 2023). This process has contributed to a documented decline in overall mental health levels over time (Xin et al., 2020; Rong et al., 2020), highlighting increasingly prominent public health hazards. Epidemiological surveys (Xin et al., 2020; Rong et al., 2020; Yang et al., 2023) reveal that the co-occurrence rate of depression and anxiety among older adults globally ranges from 20 to 30%. In China, the detection rates of anxiety and depression are 22.1% (16.8–27.2%) and 22.6% (6.3–53.6%), respectively, indicating that mental health issues such as depression and anxiety have become serious threats to the health of older adults.

Research on non-pharmacological interventions for mental health, including exercise therapy and environmental psychology approaches, has expanded substantially. Numerous cross-sectional, longitudinal, and interventional studies (Wooller et al., 2018; He et al., 2020; Iwon et al., 2021; Pang and Kim, 2021; Ji et al., 2022; Gao et al., 2023) have confirmed that physical activities differing in type, frequency, and intensity can improve mental health to varying degrees. These benefits include reducing depression and anxiety, alleviating stress, enhancing well-being and self-esteem, and improving short-term emotions (He et al., 2020). The evidence base spans from correlational analyses to causal inference, establishing exercise as a robust non-pharmacological strategy. Concurrently, research in environmental psychology (He et al., 2020; de Brito et al., 2020; Gerdes et al., 2022; Gao et al., 2023) has demonstrated associations between environmental characteristics (e.g., type, composition, proximity, and scale) and mental health outcomes such as depression, anxiety, and stress. Different environmental configurations have been shown to mitigate negative emotions, with effect sizes varying according to spatial attributes. Interdisciplinary investigations (de Brito et al., 2019; Rogerson et al., 2020; He et al., 2020; Gao et al., 2023) highlight the emerging paradigm of “green exercise” and “blue exercise,” whereby physical activity conducted in green and blue environments yields greater mental health benefits than exercise or environmental exposure alone, a phenomenon termed the “synergistic effect.” This framework has gained increasing attention in public health due to its potential to integrate the restorative properties of nature with the psychological benefits of exercise. However, synthesis of the literature reveals inconsistent findings. Early studies (Van den Berg et al., 2003) favored green environments for emotional improvement, although these investigations often included limited blue elements (e.g., small water bodies with low esthetic quality). In contrast, subsequent research (Völker and Kistemann, 2015; Wheeler et al., 2015; Gao et al., 2023) suggests that blue environments may be more effective, particularly in studies emphasizing water visibility and esthetic appeal. Correlational and subjective assessment studies (Völker and Kistemann, 2015; Wheeler et al., 2015) further support the superior emotional benefits of blue environments. In addition, the “compensation effect” theory (He et al., 2020; Gao et al., 2023) proposes that individuals with high exposure to green environments may derive greater mental health benefits from blue exercise, and vice versa, suggesting a dynamic interplay between different environmental types.

Tai Chi, a traditional Chinese mind–body practice emphasizing the “simultaneous cultivation of form and spirit,” integrates physical movement with mental tranquility. Its core principles, namely, relaxation, stillness, emptiness, and spirituality, underscore the primacy of mental calmness over physical exertion. Practitioners are guided to achieve states of “voidness and tranquility” and “modesty and desirelessness,” in which mental stillness serves as the foundation for physical movement (Kang et al., 2024). As a non-pharmacological intervention, Tai Chi has been increasingly applied to reduce negative emotions and improve mental health in older adults. Substantial evidence (Chao et al., 2014; Ji, 2018; Hua and Sun, 2021) demonstrates that Tai Chi exercise: (1) alleviates depressive, anxious, and tense states; (2) mitigates emotional fluctuations and enhances overall emotional well-being; and (3) improves heart rater variability (HRV) parameters, including total power and normalized high-frequency (HF) power, which are physiological markers of mental health.

Based on the above analysis, it is hypothesized that combining Tai Chi—a “mind–body exercise” (Hua and Sun, 2021)—with green and blue environment exposure as a “dual intervention” will yield superior mental health benefits compared with single Tai Chi or environmental exposure interventions. This synergistic approach is expected to demonstrate more pronounced effects in promoting mental well-being. Given that most existing studies use “emotion” as a composite indicator of short-term changes in mental health (e.g., depression, anxiety, stress, and burnout; Khoury et al., 2015; Hlubocky et al., 2016; Rautio et al., 2018), the present study adopts emotional indices to assess mental health in elderly males. Using an acute longitudinal dual-intervention model of “short-term green and blue exposure + short-term Tai Chi exercise + short-term continuous green and blue exposure,” this study aims to: (1) investigate the effects of acute green and blue environment exposure combined with Tai Chi exercise on the short-term emotions of elderly males; (2) comparatively analyze the characteristics of short-term emotional changes resulting from acute exposure to conventional environments, green environments, blue environments, and balanced green-blue environments, each paired separately with Tai Chi exercise; and (3) evaluate differences in the maintenance of short-term emotional effects following subsequent acute exposure to different environments after the combined intervention. This research seeks to: (1) enrich domestic literature by providing empirical evidence for green and blue exercise in mental health promotion and by expanding the research paradigm of Tai Chi in mental health; and (2) inform public health policy formulation, urban built environment planning, and landscape design.

## Materials and methods

### Participants

Inclusion criteria were as follows: (1) Participants were males aged 60 years and above, confirmed through medical history inquiry and physical examination to meet the basic health requirements for participation in Tai Chi exercise and environmental exposure interventions. (2) Participants passed the 2002 revised Physical Activity Readiness Questionnaire, with no restrictions on physical activity and no symptoms such as chest tightness, chest pain, or shortness of breath. (3) Participants had no regular exercise habits and had not practiced Tai Chi or other structured fitness programs. Exclusion criteria included: (1) Individuals with high physical activity levels or those whose workplace or residence was close to the experimental site. (2) Individuals who had sustained injuries, consumed alcohol, strong tea, coffee, or other stimulating beverages, excessively consumed spicy or other irritating foods, taken psychotropic drugs or medications that may affect cardiovascular function or emotional states, or experienced significant emotional fluctuations within 24 h prior to the experiment. (3) Individuals with color blindness, severe mental disorders, severe cardiovascular or cerebrovascular diseases, motor system diseases, autoimmune diseases, tumors, or endocrine disorders, as confirmed by medical history inquiry and physical examination.

Sample size calculation was conducted using G*Power 3.1 software, with the significance level *α* set at 0.05, statistical power *β* at 0.80, and effect size f at 0.25 (Gao et al., 2023). The calculation indicated a minimum sample size of 20 participants per group. Considering a 15% attrition rate, 100 eligible participants were recruited from Qinlou Subdistrict, Donggang District, Rizhao City, Shandong Province.

A random number table method was adopted to assign participants to four groups: conventional environment + Tai Chi, green environment + Tai Chi, blue environment + Tai Chi, and balanced green-blue environment + Tai Chi. The specific procedure was as follows. First, SPSS 19.0 was used to generate a random number sequence ranging from 1 to 4, with each number pre-assigned to one of the four groups to ensure equal allocation probability. Second, eligible participants were assigned unique sequential serial numbers according to the order of recruitment. Finally, each participant’s serial number was matched with the pre-generated random number sequence to determine group allocation. Participants who withdrew during the study or exhibited abnormal test data were excluded, resulting in final sample sizes of 23, 24, 23, and 24 for the TJQ, GTJQ, BTJQ, and GBTJQ groups, respectively. Baseline characteristics did not differ significantly among groups (see Table 1).

**Table 1:** Descriptive statistics of age, height, weight and educational level among participants in each group.

Metrics		TJQ	GTJQ	BTJQ	GBTJQ
Age (years)		63.50 ± 2.31	62.42 ± 2.08	62.20 ± 3.19	63.54 ± 2.17
Height (cm)		174.20 ± 3.89	174.27 ± 4.21	173.41 ± 3.22	175.02 ± 3.01
Weight (kg)		70.12 ± 3.31	71.81 ± 3.94	69.59 ± 3.27	70.52 ± 4.02
Educational Level (%)	Junior High School or Below	8.70	12.50	4.35	12.50
	High School/Technical School	43.48	37.50	47.83	37.50
	Undergraduate/College	34.78	41.67	39.13	37.50
	Graduate or Above	13.04	8.33	8.70	12.50

TJQ, conventional environment + Tai Chi; GTJQ, green environment + Tai Chi; BTJQ, blue environment + Tai Chi; GBTJQ, balanced green-blue environment + Tai Chi.

Based on the National Physical Fitness Monitoring physical activity questionnaire, all of the participants exhibited no significant differences in daily physical activity levels. Participants were fully informed of the experimental procedures and precautions, and written informed consent was obtained from all of the participants. The study was approved by the Ethics Committee of Shandong Sport University.

### Experimental protocol

Tai Chi exercise requires a stable center of gravity, with the lower limbs primarily maintained in flexed or semi-squatting postures, thereby imposing a relatively high load. Because elderly beginners typically demonstrate limited lower-limb muscle strength, performing low-stance movements can be challenging. Therefore, this study adopted a middle–high stance Tai Chi protocol, in which knee joint angles were maintained at approximately 130–140 degrees during exercise, consistent with previous studies by Zhou et al. (2005) and Zhang and Duan (2013). Professional instructors guided participants through a 1-week Tai Chi training program accompanied by rhythmic music to ensure correct mastery of all of the movements. A 10-day washout period was implemented prior to the formal experiment to minimize residual effects of prior physical activity.

The experimental protocol was primarily based on the designs reported by He et al. (2020), Gao et al. (2023), Tyrväinen et al. (2014), and Ojala et al. (2019), adopting an acute longitudinal dual short-term intervention maintenance model: “short-term green and blue environment exposure + short-term Tai Chi exercise + continuous short-term green and blue environment exposure.” The experimental sites were located in Donggang District, Rizhao City, Shandong Province. A single-day experimental design was employed, and all of the procedures for each participant, including baseline measurement, 20-min environmental viewing, 45-min Tai Chi exercise, and post-exercise recovery monitoring until heart rate returned to resting level, were completed on the same day. The experimental period extended from mid-September to mid-October 2024, when ambient temperatures were appropriate, vegetation and seawater quality were favorable, and insect activity was minimal.

**Figure 1:**
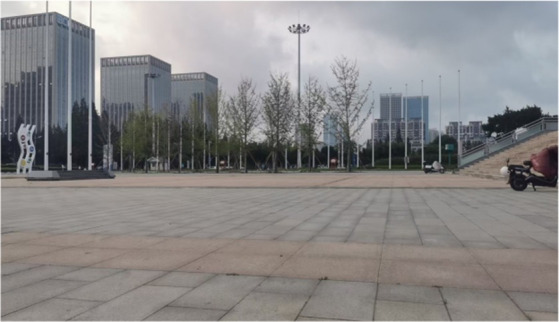
Conventional environmental landscape.

**Figure 2:**
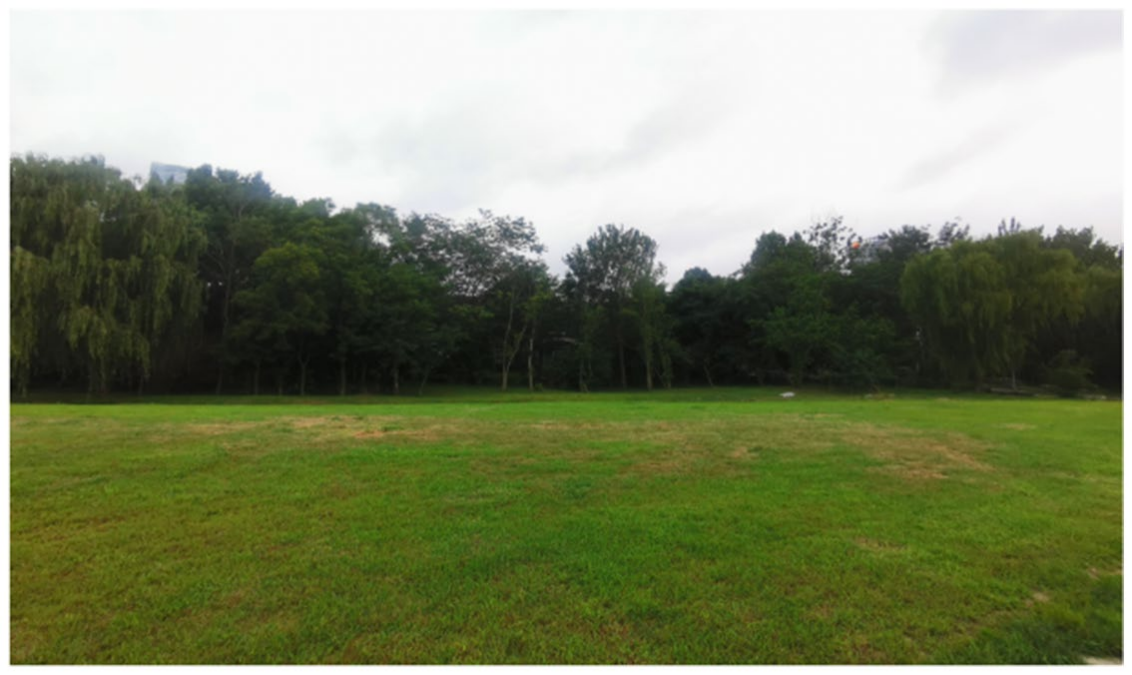
Green environmental landscape.

**Figure 3:**
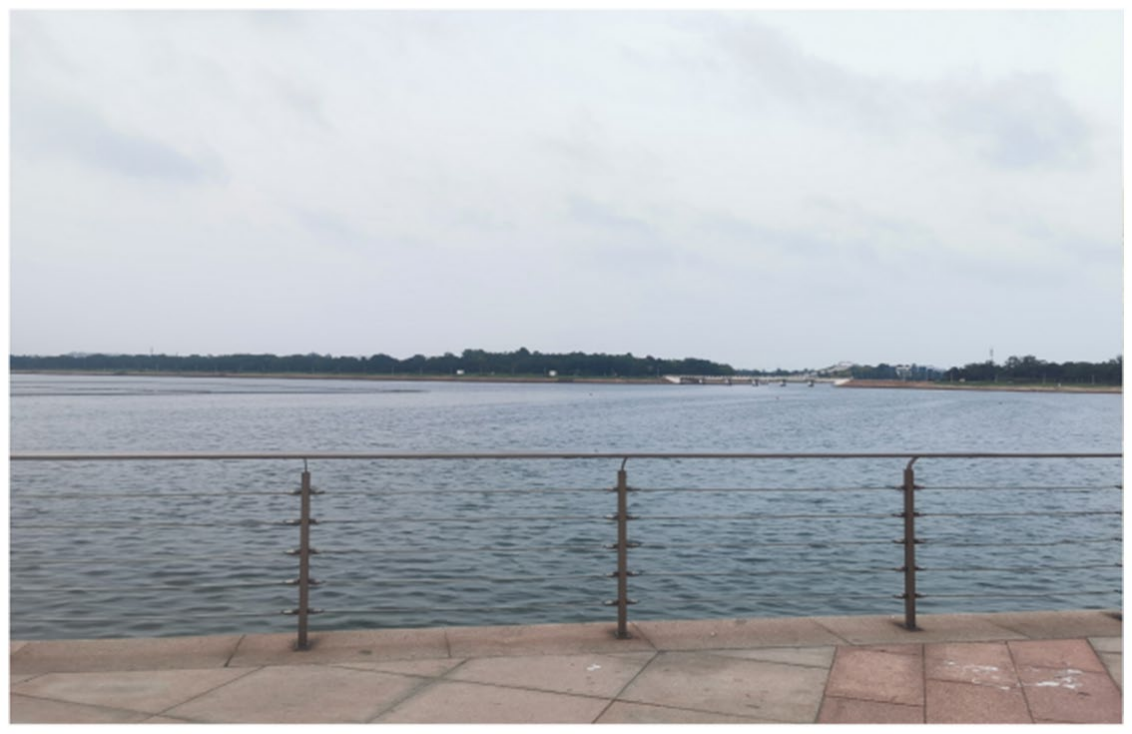
Blue environmental landscape.

**Figure 4:**
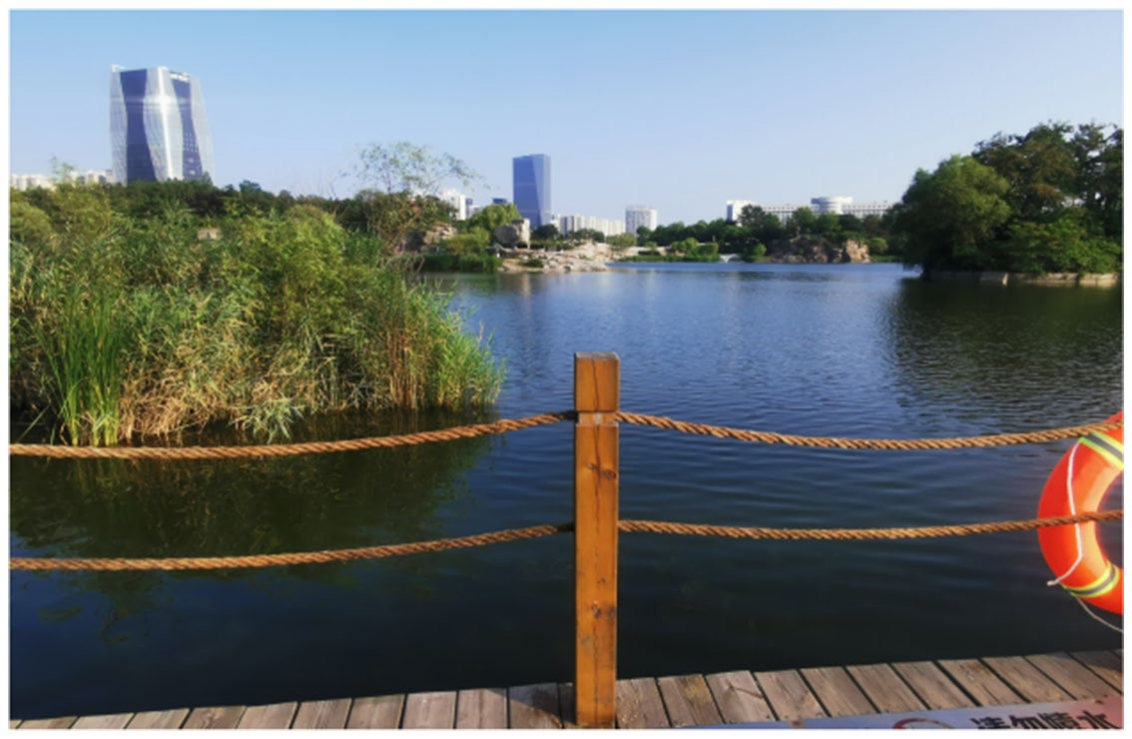
Green-blue environmental balanced landscape.

**Figure 5:**
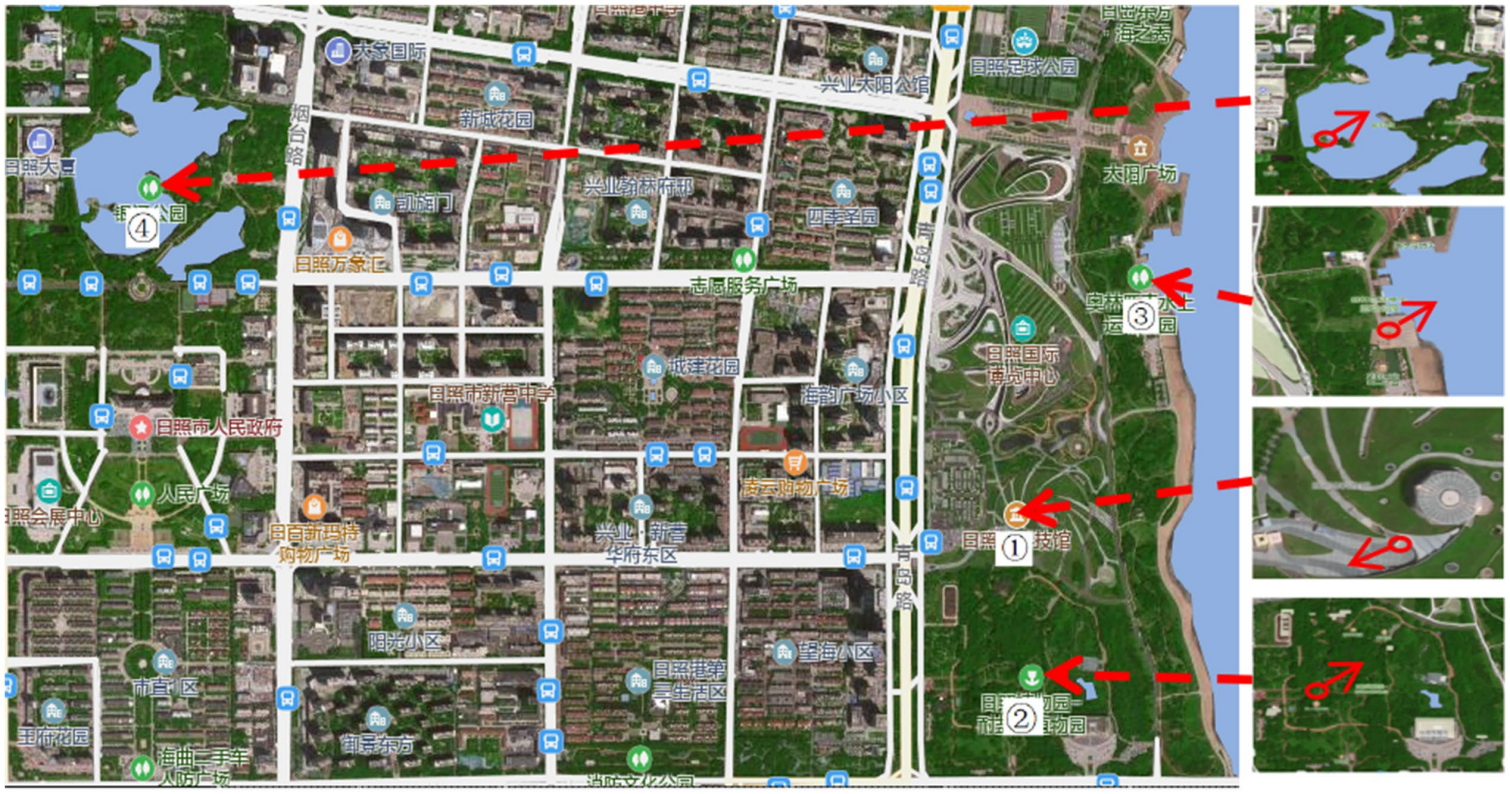
Satellite map of experimental sites. (1) ①: TJQ (Rizhao Science and Technology Museum); ②: GTJQ (Rizhao Botanical Garden); ③: BTJQ (Rizhao Olympic Water Sports Park); ④: GBTJQ (Rizhao Yinhe Park). (2) →: Viewing direction of environmental space. (3) This figure is from https://map.bmcx.com/rizhao__map/.

Four landscape viewing sites corresponding to the different experimental conditions were established. All of the sites were selected using back-to-back criteria to ensure comparable visual openness. The characteristics of each site were as follows. TJQ: The viewing site was located at Rizhao Science and Technology Museum, an artificially constructed marble square with extremely low natural vegetation coverage (Figure 1). GTJQ: The viewing site was located at Rizhao Botanical Garden, characterized by abundant green plants with dense and diverse vegetation (Figure 2). BTJQ: The viewing site was located at Rizhao Olympic Water Sports Park, a typical coastal waterfront area adjacent to the shoreline, featuring expansive open water surfaces and coastal views (Figure 3). GBTJQ: The viewing site was located at Rizhao Yinhe Park, where waterfront areas were organically integrated with extensive vegetation coverage (Figure 4). All four landscape sites were situated within the central urban area of Rizhao, with inter-site distances of less than 10 km. This site selection strategy helped control for potential confounding effects of commuting time on participants’ physical and emotional states. The geographic locations and spatial distribution of the four sites are illustrated in the satellite map (Figure 5), with the corresponding site numbers as follows: ① TJQ (Rizhao Science and Technology Museum), ② GTJQ (Rizhao Botanical Garden), ③ BTJQ (Rizhao Olympic Water Sports Park), and ④ GBTJQ (Rizhao Yinhe Park).

Participants arrived at the testing site at 8:00 a.m. or 9:00 a.m. and completed all of the procedures in a single session according to their scheduled time slots. The protocol was implemented as follows. First, participants wore a heart rate monitor and rested in a seated position for 3 min. During this period, HRV, blood pressure, and subjective emotional indices were collected, while environmental temperature, humidity, and noise decibels were recorded simultaneously. Second, after a 20-min seated rest, participants viewed the designated landscape spaces, after which HRV, blood pressure, emotional indices, and environmental parameters were re-measured. Participants then performed a 45-min middle–high stance Tai Chi exercise guided by rhythmic accompaniment. Immediately after exercise, HRV, blood pressure, emotional indices, and environmental factors were measured at the rest station. Finally, participants continued landscape viewing in a seated position until heart rate returned to baseline, at which point final measurements of HRV, blood pressure, emotional indices, and ambient conditions were recorded.

**Figure 6:**
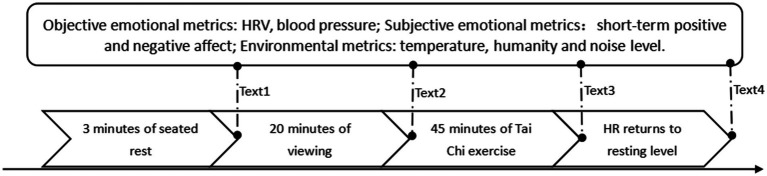
Experimental flowchart. Test 1: Prior to the experiment; Test 2: 20 min after environmental landscape viewing; Test 3: Immediately after Tai Chi exercise; Test 4: When heart rate recovered to resting level.

Participants were instructed to maintain their usual lifestyle routines from the beginning of Tai Chi training until the completion of the experiment, with specific restrictions including avoidance of any additional exercise programs and avoidance of exposure to adverse mental stimuli. To ensure safety, participants were permitted to stand for 1–2 min in front of the rest station after Tai Chi exercise, while continuously viewing their assigned landscape space. Immediately after exercise and prior to seated recovery, participants were provided with a 380 mL bottle of Nongfu Spring mineral water, which could be consumed at their discretion (Figure 6).

## Assessments

### Objective emotional metrics

*HRV Index Test*: Referring to the methods of He et al. (2020) and Gao et al. (2023), the Firstbeat Sports heart rate monitoring system (Model: Polar H10 Heart Rate Sensor; Manufacturer: Polar Electro Oy) was used to collect HRV data throughout the experiment. The primary indicators included the standard deviation of normal-to-normal R–R intervals (SDNN), the root mean square of successive differences (rMSSD), and the ratio of low frequency (LF) to high frequency (HF) power (LF/HF). According to the experimental protocol, HRV data were sampled at the following time points: (1) prior to the experiment (Test 1), using SDNN, rMSSD, and LF/HF values within 0–5 min; (2) 20 min after environmental landscape viewing (Test 2), using values from 15–20 min following the 20-min viewing period; (3) immediately after Tai Chi exercise (Test 3), using values from 60–65 min; and (4) post-recovery (Test 4), using values from the final 5 min before heart rate returned to the resting level.

*Blood Pressure Index Measurement*: Blood pressure was measured using an OMRON HEM-7200 electronic sphygmomanometer (Manufacturer: Omron Healthcare Co., Ltd.) on the right upper arm of each participant. Participants remained seated with the elbow positioned at heart level. Following the procedures described by Gao et al. (2023) and Ojala et al. (2019), two consecutive measurements were obtained, and the mean value was recorded. Blood pressure assessments were conducted at four stages: prior to the experiment (Test 1), 20 min after environmental landscape viewing (Test 2), immediately after Tai Chi exercise (Test 3), and when heart rate recovered to the resting level (Test 4).

### Subjective emotional metrics

*Short-Term Positive and Negative Affect Assessment*: The Positive and Negative Affect Schedule Short-Form (PANAS-SF), an internationally validated instrument, was employed to assess short-term positive and negative affect (Thompson, 2007). The Chinese version of the PANAS-SF demonstrates good reliability and validity (Liu et al., 2015) and consists of 10 items, including five positive affect items reflecting active, pleasant, and energetic experiences, and five negative affect items reflecting unpleasant, anxious, and depressed states. Responses were rated on a 5-point Likert scale, with total scores for positive affect and negative affect ranging from 5 to 25. Higher scores indicate stronger positive or negative emotions, respectively. Short-term positive and negative affect were assessed at four stages: prior to the experiment (Test 1), 20 min after environmental landscape viewing (Test 2), immediately after Tai Chi exercise (Test 3), and when heart rate recovered to the resting level (Test 4).

### Environmental metrics

*Temperature and Humidity Measurement*: Environmental temperature and humidity were measured using a HuaTu A2000-TH thermohygrometer (Manufacturer: Shenzhen HUATO System Co., Ltd.).

*Noise Level Measurement*: Ambient noise levels were measured using a DeLi DL333201 decibel meter (Manufacturer: DELI Group Co., Ltd.).

Environmental metrics were recorded at four stages: prior to the experiment (Test 1), 20 min after environmental landscape viewing (Test 2), immediately after Tai Chi exercise (Test 3), and when heart rate recovered to the resting level (Test 4).

Considering the cumulative effects of environmental factors on human physiology, and following the approach of Gao et al. (2023), mean values of environmental indicators across the different experimental time points were analyzed. No significant differences were observed among the TJQ, GTJQ, BTJQ, and GBTJQ groups, as shown in Table 2.

**Table 2:** Descriptive statistics of temperature, humidity, and noise at experimental sites.

Metrics	TJQ	GTJQ	BTJQ	GBTJQ
Temperature (°C)	22.43 ± 3.62	22.34 ± 3.09	20.87 ± 4.02	21.52 ± 2.87
Humidity (%RH)	51.58 ± 8.94	52.46 ± 10.24	53.60 ± 9.43	53.07 ± 9.87
Noise Level (dBA)	52.34 ± 2.01	51.23 ± 3.10	50.64 ± 4.54	51.34 ± 3.27

TJQ, conventional environment + Tai Chi; GTJQ, green environment + Tai Chi; BTJQ, blue environment + Tai Chi; GBTJQ, balanced green-blue environment + Tai Chi.

### Statistical analysis

Data analysis was conducted using SPSS 19.0 software, and all of the measurement data were expressed as mean ± standard deviation (Mean ± SD). Descriptive statistical analyses were performed for dependent variables and socio-demographic characteristics. Additional quantitative statistical procedures were as follows.

(1) Comparison among Test 1, Test 2, Test 3, and Test 4: Repeated measures analysis of variance (ANOVA) was used to examine differences in SDNN, rMSSD, LF/HF ratio, systolic blood pressure (SBP), diastolic blood pressure, and PANAS-SF positive affect scores across the four time points within each group to evaluate short-term emotional improvement effects. Because PANAS-SF negative affect scores did not conform to a normal distribution, non-parametric tests were applied to compare negative affect scores across the four time points within each group. A *p*-value < 0.05 was considered to be statistically significant.
(2) Inter-group and intra-group comparisons: Repeated measures ANOVA was applied to compare short-term emotional improvement effects between groups at the same test time point and within groups across different test time points. *Post-hoc* tests were conducted for pairwise comparisons to analyze differential effects of combined exposure to different environments and Tai Chi exercise on HRV, blood pressure, positive affect, and negative affect. Statistical significance was set at *p* < 0.05, and partial eta-squared (ɳ2p) was used as the effect size indicator.
(3) HRV data processing: For HRV analysis, 5-min heart rate segments recorded at different experimental phases by the Firstbeat Sports software were imported into Excel 2010. Corrected R–R intervals were applied, after which time-domain and frequency-domain analysis tables were generated and exported.

## Results

This study was an acute intervention study that adopted an acute longitudinal dual-effect model of “20-min acute environmental exposure + 45-min acute Tai Chi exercise + short-term continuous environmental exposure” to examine the acute effects of the combined intervention on the emotions of elderly males and the maintenance of these effects during post-exercise recovery. All of the data were collected at four time points within a single acute intervention session (prior to the experiment, 20 min after environmental landscape viewing, immediately after Tai Chi exercise, and when heart rate recovered to resting level), and the results reflect short-term physiological and emotional response characteristics under acute intervention conditions, as follows.

### Objective emotional metrics of participants across groups

As shown in Supplementary Table 1, SDNN and rMSSD values in the TJQ, GTJQ, BTJQ, and GBTJQ groups showed a decreasing trend during the acute experimental phase and an increasing trend during the recovery phase. In contrast, LF/HF ratios increased during the acute experimental phase and decreased during the recovery phase. For intra-group comparisons across time points, relative to baseline (Test 1), (1) at 20 min post-landscape viewing (Test 2), the BTJQ group showed significantly lower SDNN (ɳ2p = 0.162, *p* = 0.045) and rMSSD (ɳ2p = 0.137, *p* = 0.046); (2) immediately after Tai Chi exercise (Test 3), the GTJQ group exhibited significant decreases in SDNN (ɳ2p = 0.112, *p* = 0.048) and rMSSD (ɳ2p = 0.154, *p* = 0.047), while the BTJQ and GBTJQ groups showed highly significant decreases in SDNN (ɳ2p = 0.284, *p* = 0.005; ɳ2p = 0.202, *p* = 0.007) and rMSSD (ɳ2p = 0.365, *p* = 0.001; ɳ2p = 0.275, *p* = 0.006); and (3) during full recovery (Test 4), the BTJQ group still showed significantly lower SDNN (ɳ2p = 0.137, *p* = 0.042) than baseline. Compared with immediately post-exercise (Test 3), the BTJQ group exhibited a significant increase in SDNN (ɳ2p = 0.158, *p* = 0.044) during recovery (Test 4), indicating partial physiological recovery following the acute intervention.

For inter-group comparisons at the same time points, relative to the TJQ group, (1) at 20 min post-landscape viewing (Test 2), the BTJQ group showed significantly lower SDNN (ɳ2p = 0.159, *p* = 0.049) and rMSSD (ɳ2p = 0.161, *p* = 0.047), and the GBTJQ group showed a significant decrease in rMSSD (ɳ2p = 0.178, *p* = 0.043); (2) immediately after Tai Chi exercise (Test 3), the BTJQ group had significantly lower SDNN (ɳ2p = 0.278, *p* = 0.015) and rMSSD (ɳ2p = 0.415, *p* = 0.001), and both the GTJQ and GBTJQ groups showed significant reductions in rMSSD (ɳ2p = 0.136, *p* = 0.049; ɳ2p = 0.190, *p* = 0.026); and (3) during full recovery (Test 4), the BTJQ group still showed significantly lower SDNN (ɳ2p = 0.126, *p* = 0.028). Relative to the GTJQ group, (1) at 20 min post-landscape viewing (Test 2), the BTJQ group showed a significant decrease in SDNN (ɳ2p = 0.187, *p* = 0.028); (2) immediately after Tai Chi exercise (Test 3), the BTJQ group showed significant reductions in SDNN (ɳ2p = 0.212, *p* = 0.022) and rMSSD (ɳ2p = 0.302, *p* = 0.012); and (3) during full recovery (Test 4), the BTJQ group exhibited significantly lower SDNN (ɳ2p = 0.126, *p* = 0.044).

**Figure 7:**
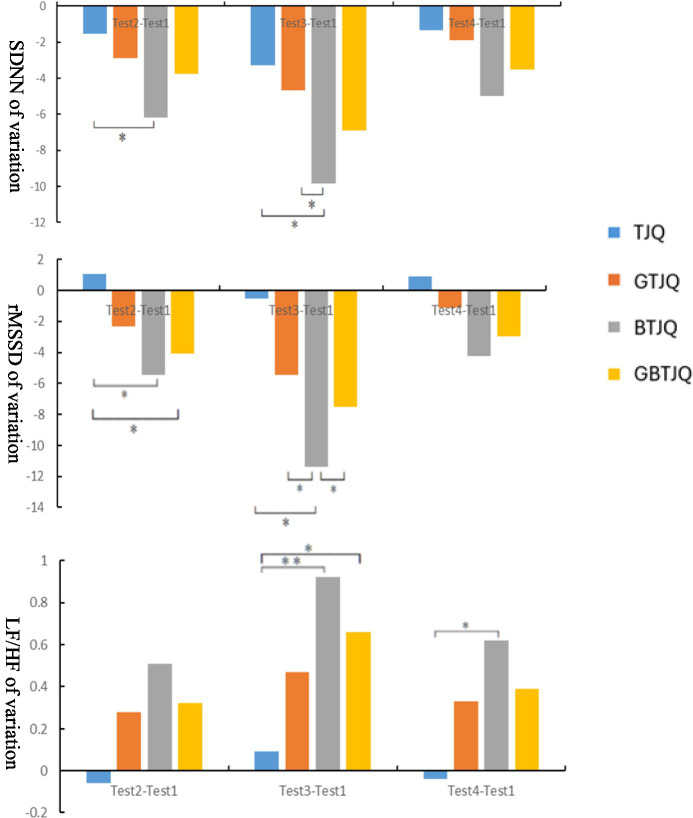
Inter-group comparisons of HRV metrics’ variations across measurement time points. (1) * indicates *p* < 0.05, denoting a statistically significant difference; ** indicates *p* < 0.01, denoting a highly statistically significant difference. (2) Test 1: prior to the experiment; Test 2: 20 min after environmental landscape viewing; Test 3: immediately after Tai Chi exercise; Test 4: when heart rate recovered to resting level. (3) TJQ, conventional environment + Tai Chi; GTJQ, green environment + Tai Chi; BTJQ, blue environment + Tai Chi; GBTJQ, balanced green-blue environment + Tai Chi. (4) SDNN, the standard deviation of normal-to-normal R–R intervals; rMSSD, root mean square of successive differences; LF/HF, the ratio of LF to HF.

Figure 7 presents inter-group comparisons of the magnitudes of within-group changes in each HRV indicator at different time points relative to baseline (Test 1). Across the three post-baseline time points—20 min post-landscape viewing (Test 2), immediately after Tai Chi exercise (Test 3), and during full recovery (Test 4)—the inter-group comparisons of the magnitudes of change in SDNN, rMSSD, and LF/HF followed the order: BTJQ > GBTJQ > GTJQ > TJQ. At 20 min post-landscape viewing (Test 2), changes in SDNN and rMSSD differed significantly between the TJQ and BTJQ groups (*p* < 0.05), and changes in rMSSD differed significantly between the TJQ and GBTJQ groups (*p* < 0.05). Immediately after Tai Chi exercise (Test 3), changes in SDNN, rMSSD, and LF/HF differed significantly between the TJQ and BTJQ groups (*p* < 0.05), changes in SDNN and rMSSD differed significantly between the GTJQ and BTJQ groups (*p* < 0.05), changes in rMSSD differed significantly between the BTJQ and GBTJQ groups (*p* < 0.05), and changes in LF/HF differed significantly between the TJQ and GBTJQ groups (*p* < 0.05). During full recovery (Test 4), changes in LF/HF differed significantly between the TJQ and BTJQ groups (*p* < 0.05).

Supplementary Table 2 shows that SBP in the TJQ, GTJQ, BTJQ, and GBTJQ groups decreased at 20 min post-landscape viewing (Test 2), increased immediately after Tai Chi exercise (Test 3), and decreased again during full recovery (Test 4). Diastolic blood pressure (DBP) showed a decreasing trend during the acute experimental phase and an increasing trend during full recovery. For intra-group comparisons across time points, relative to baseline (Test 1), (1) at 20 min post-landscape viewing (Test 2), the BTJQ group showed significant decreases in both SBP (ɳ2p = 0.085, *p* = 0.046) and DBP (ɳ2p = 0.106, *p* = 0.044); (2) immediately after Tai Chi exercise (Test 3), the TJQ group showed a significant increase in SBP (ɳ2p = 0.168, *p* = 0.049), while the BTJQ and GBTJQ groups showed significantly lower DBP (ɳ2p = 0.185, *p* = 0.027; ɳ2p = 0.082, *p* = 0.049); and (3) during full recovery (Test 4), the BTJQ group still showed significantly lower SBP (ɳ2p = 0.092, *p* = 0.041) than baseline. Relative to 20 min post-landscape viewing (Test 2), the TJQ and GTJQ groups showed significant increases in SBP (ɳ2p = 0.233, *p* = 0.011; ɳ2p = 0.173, *p* = 0.047) immediately after Tai Chi exercise (Test 3). Relative to immediately after Tai Chi exercise (Test 3), the TJQ and GTJQ groups showed significant decreases in SBP (ɳ2p = 0.221, *p* = 0.015; ɳ2p = 0.136, *p* = 0.037) during full recovery (Test 4).

For inter-group comparisons at the same time points, no significant differences in SBP or DBP were observed among the TJQ, GTJQ, BTJQ, and GBTJQ groups at any measurement time point.

**Figure 8:**
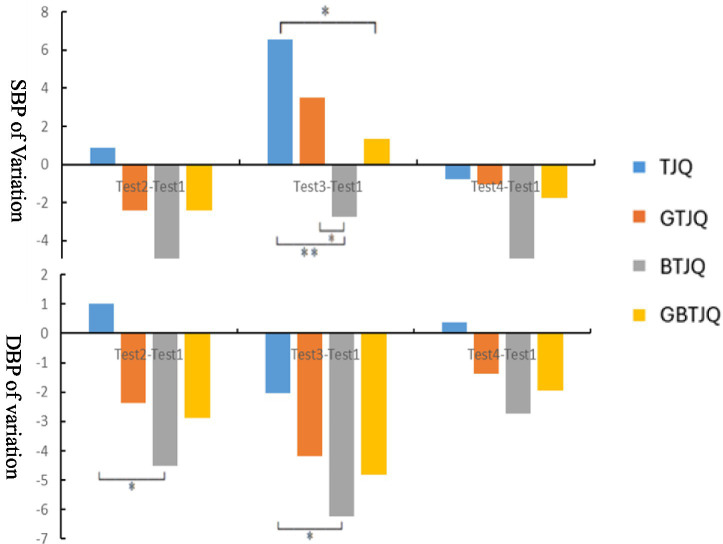
Inter-group comparisons of blood pressure metrics’ variations across measurement time points. (1) * Indicates *p* < 0.05, denoting a statistically significant difference; ** indicates *p* < 0.01, denoting a highly statistically significant difference. (2) Test 1: prior to the experiment; Test 2: 20 min after environmental landscape viewing; Test 3: immediately after Tai Chi exercise; Test 4: when heart rate recovered to resting level. (3) TJQ, conventional environment + Tai Chi; GTJQ, green environment + Tai Chi; BTJQ, blue environment + Tai Chi; GBTJQ, balanced green-blue environment + Tai Chi. (4) SBP, ystolic blood pressure; DBP, diastolic blood pressure.

Figure 8 illustrates inter-group comparisons of the magnitudes of within-group changes in SBP and DBP at different time points relative to baseline (Test 1). The inter-group comparisons of the magnitude of SBP change followed the order BTJQ > GBTJQ > GTJQ > TJQ at 20 min post-landscape viewing (Test 2) and during full recovery (Test 4), whereas immediately after Tai Chi exercise (Test 3) the order was TJQ > GTJQ > BTJQ > GBTJQ. The inter-group comparisons of the magnitude of DBP change followed the order BTJQ > GBTJQ > GTJQ > TJQ at 20 min post-landscape viewing (Test 2), immediately after Tai Chi exercise (Test 3), and during full recovery (Test 4). At 20 min post-landscape viewing (Test 2), SBP and DBP changes differed significantly between the TJQ and BTJQ groups (*p* < 0.05). Immediately after Tai Chi exercise (Test 3), SBP changes differed significantly between the TJQ and BTJQ groups (*p* < 0.05) and between the TJQ and GBTJQ groups (*p* < 0.05), and DBP changes differed significantly between the GTJQ and BTJQ groups (*p* < 0.05). During full recovery (Test 4), SBP changes differed significantly between the TJQ and BTJQ groups (*p* < 0.05).

### Subjective emotional metrics of participants across groups

As shown in Supplementary Table 3, positive affect scores in the TJQ, GTJQ, BTJQ, and GBTJQ groups increased during the acute experimental phase and decreased during the recovery phase. In contrast, negative affect scores showed a decreasing trend during the acute experimental phase and continued to decrease during the recovery phase. Notably, no significant differences in positive or negative affect scores were observed within groups across time points (intra-group temporal comparisons) or between groups at the same time point (inter-group comparisons).

**Figure 9:**
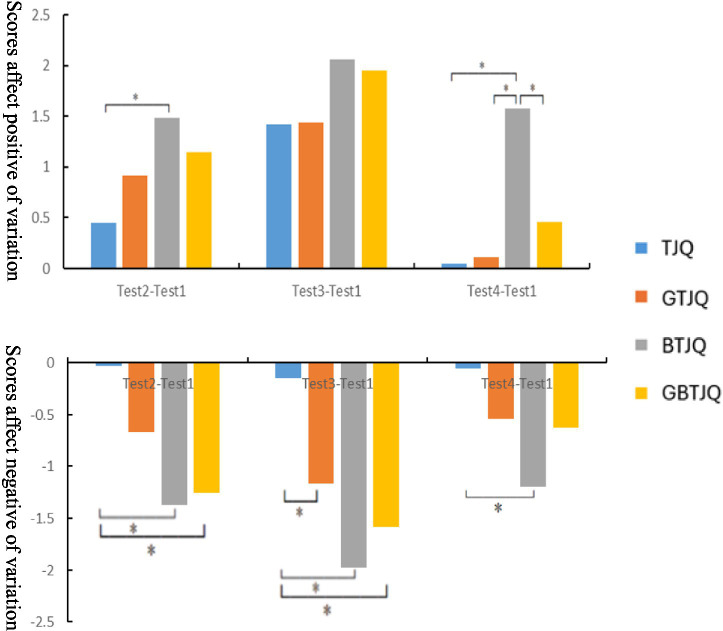
Inter-group comparisons of subjective emotional metrics’ variations across measurement time points. (1) * indicates *p* < 0.05, denoting a statistically significant difference; ** indicates *p* < 0.01, denoting a highly statistically significant difference. (2) Test 1: Rior to the experiment; Test 2: 20 min after environmental landscape viewing; Test 3: immediately after Tai Chi exercise; Test 4: when heart rate recovered to resting level. (3) TJQ, conventional environment + Tai Chi; GTJQ, green environment + Tai Chi; BTJQ, blue environment + Tai Chi; GBTJQ, balanced green-blue environment + Tai Chi.

Figure 9 presents inter-group comparisons of the magnitudes of within-group changes in positive and negative affect scores at different time points relative to baseline (Test 1). Across all of the post-baseline time points—20 min post-landscape viewing (Test 2), immediately after Tai Chi exercise (Test 3), and during full recovery (Test 4)—The inter-group comparisons of the magnitudes of change in both positive affect and negative affect followed the order: BTJQ > GBTJQ > GTJQ > TJQ. At 20 min post-landscape viewing (Test 2), changes in positive and negative affect differed significantly between the TJQ and BTJQ groups (*p* < 0.05), and changes in negative affect differed significantly between the TJQ and GBTJQ groups (*p* < 0.05). Immediately after Tai Chi exercise (Test 3), changes in negative affect differed significantly between the TJQ and GTJQ groups (*p* < 0.05), between the TJQ and BTJQ groups (*p* < 0.05), and between the TJQ and GBTJQ groups (*p* < 0.05). During full recovery (Test 4), changes in positive and negative affect differed significantly between the TJQ and BTJQ groups (*p* < 0.05), and changes in positive affect differed significantly between the GTJQ and BTJQ groups (*p* < 0.05) and between the BTJQ and GBTJQ groups (*p* < 0.05).

## Discussion

This study focuses on an acute intervention scenario and employed a single-day, one-time combined intervention of environmental exposure and Tai Chi to examine acute effects and post-recovery maintenance patterns in the emotional health of elderly males. Unlike long-term intervention studies (e.g., longitudinal follow-up and cumulative effects of repeated interventions), this study emphasizes the acute effect chain of “single intervention → immediate response → short-term maintenance,” and the findings provide a practical basis for short-term emotional regulation and immediate psychological intervention. The discussion below integrates evidence from similar acute intervention studies, with emphasis on comparing differences in effects and similarities in underlying mechanisms under a single intervention.

### Analysis of the acute effects of green and blue environment exposure on short-term emotions in elderly males

The results indicated that, compared with baseline, the GTJQ, BTJQ, and GBTJQ groups showed favorable changes in HRV, blood pressure, and short-term emotional states after 20-min viewing of green and blue landscapes. Notably, the BTJQ group showed favorable changes in SDNN, rMSSD, and SBP. Furthermore, relative to baseline, the BTJQ group demonstrated the largest favorable changes in SDNN, rMSSD, LF/HF, SBP, DBP, positive affect, and negative affect after 20-min landscape viewing, followed by the GBTJQ, GTJQ, and TJQ groups (least improvement). Significant differences in the magnitudes of favorable changes were observed between the TJQ and BTJQ groups for SDNN, rMSSD, blood pressure, and affect scores, and between the TJQ and GBTJQ groups for rMSSD and negative affect. These findings indicate that environmental exposure types differ in their acute emotional benefits, with the following ranking: blue environment > balanced green-blue environment > green environment > conventional environment.

These findings are consistent with Wooller et al. (2018), who used an acute intervention of “single simulated natural environment video viewing + moderate-intensity exercise” and reported that short-term exposure to natural environments can rapidly reduce acute psychological stress in adults, with emotional improvement occurring immediately after the intervention. Rogerson et al. (2016a, 2016b) compared indoor and outdoor aerobic cycling in adults aged 18–73 years and park running in adults (40.8 ± 12.00 years) and reported that outdoor cycling was more effective for emotional improvement; running by rivers, beaches, or park grasslands also alleviated stress and improved mood compared with pre-exercise states, which aligns with the present conclusion regarding the superior acute effect of blue environments.

Accumulating evidence from multiple study designs supports the association between green and blue environment exposure and improved mental health. Cross-sectional studies indicate that walkable, high-quality green and blue landscapes in neighborhoods are associated with lower depression levels in older adults (Berke et al., 2007). Conversely, older adults living in areas lacking high-quality green and blue landscapes, particularly in residential areas with high mixed land use (commercial, retail, industrial, etc.), show higher levels of depression and anxiety (Saarloos et al., 2011). Domestic cross-sectional evidence similarly suggests that older adults in neighborhoods with higher vegetation uniformity index and water normalization index exhibit lower depression scores (Helbich et al., 2019). Longitudinal studies provide stronger temporal evidence. Cohen-Cline et al. (2015) matched 4,338 twin adults into high and low green environment exposure groups, followed them for 6 years (2008–2014), and found that participants in high-exposure areas with higher-quality green landscapes had significantly better mental health than those in low-exposure areas. Studies in the United Kingdom with 12-year and 17-year follow-up periods similarly reported positive associations between higher-quality green and blue environments and mental health in older adults (Alcock et al., 2014; de Keijzer et al., 2019). A 5-year follow-up of 24,945 individuals in Sweden found that higher residential green environment quality was associated with better mental health, with the association strengthening with age; adults aged 63–81 years without green environments were more likely to experience depression and reduced well-being than those aged 18–38 years, potentially because neighborhoods constitute the primary living space for older adults and may increase sensitivity to green and blue landscape characteristics (Annerstedt et al., 2012). Although green and blue exposure studies cannot fully replicate randomized controlled trials due to constraints of natural environments, intervention studies provide supportive evidence of beneficial mental health effects. Roe and Aspinall (2011) reported that rural green environment walking improved mood more than urban green environment walking in 123 adults, with larger improvements observed among individuals with poorer baseline mental health, irrespective of setting.

Existing evidence on the effects of green and blue environments on mental health in older adults—from early compensation effect theories (He et al., 2020; Gao et al., 2023) to more recent cross-sectional and longitudinal “causal” studies (Gascon et al., 2015; Foley and Kistemann, 2015; de Vries et al., 2016)—consistently indicates that blue environments, when characterized by adequate water area, sanitation, water quality, and visibility, outperform green environments in promoting mental health. This advantage may be related to cortical visual area sensitivity, whereby blue and yellow hues are processed more efficiently than green and red hues, thereby facilitating mental recovery (Mullen and Kingdom, 2002). A meta-analysis further confirmed that the most pronounced mental health benefits are associated with exposure to rivers, lakes, and seas (Barton and Pretty, 2010). As an acute intervention study, the mechanism underlying mood improvement following acute environmental exposure in the present research may be related to the immediacy of sensory stimulation. Visual elements (e.g., open water surfaces and green vegetation) and auditory stimuli (e.g., flowing water and bird songs) in blue and green environments can rapidly activate the parasympathetic nervous system, reduce heart rate and blood pressure, and promote physiological relaxation (Gianfredi et al., 2021). This immediate physiological regulation is directly translated into short-term positive emotional changes, which is fundamentally different from cumulative effects that depend on long-term exposure, such as those observed in the 5-year follow-up study by Annerstedt et al. (2012). The acute effect findings of this study therefore supplement existing evidence on emotional responses of older adults to short-term environmental exposure and enhance the population applicability of acute green and blue exposure research.

### Analysis of the acute synergistic effects of green and blue environment exposure combined with tai chi exercise on short-term emotions in elderly males

The results of this study showed that, compared with baseline and 20-min environmental landscape viewing, HRV, blood pressure, and short-term emotional indices in the TJQ, GTJQ, BTJQ, and GBTJQ groups exhibited significantly positive changes immediately after Tai Chi exercise. Short-term green and blue environment exposure followed by Tai Chi exercise further amplified positive emotional changes in elderly males. Moreover, the findings indicated that the BTJQ group demonstrated the greatest magnitude of positive emotional change immediately after Tai Chi exercise, followed by the GBTJQ, GTJQ, and TJQ groups (with the smallest improvement). These results suggest that all combinations of environmental exposure and Tai Chi exercise exert positive effects on short-term emotions in elderly males, with the effectiveness ranking as follows: BTJQ > GBTJQ > GTJQ > TJQ. This pattern is consistent with the findings of Rogerson and Barton (2015), who reported that combining treadmill exercise with exposure to projected natural environment videos (forests and lakes) or urban street scenes improved stress and mood more effectively than treadmill exercise alone in adults (mean age 27.8 years), thereby confirming the synergistic effects of acute environmental stimulation and exercise. Randomized controlled trials conducted in real natural environments further demonstrated that 77 adults (mean age 47.6 years) who engaged in 15-min seated observation followed by 30-min walking in urban street green spaces, urban green parks, or urban forest parks showed significantly greater stress reduction and mental fatigue relief after walking than after seated observation alone (Tyrväinen et al., 2014). Among these settings, urban forest parks produced particularly strong mental health benefits, consistent with the present finding of the strongest acute effect immediately after exercise.

Psychological research has indicated that theories such as the self-efficacy hypothesis and attention transfer hypothesis, together with stress reduction theory, the biophilia hypothesis, and the environmental self-regulation hypothesis, can explain the positive effects of exercise and green and blue environmental exposure on mental health (He et al., 2020; He, 2020). At present, a growing body of research interprets the mechanisms through which exercise and green and blue environmental exposure improve mental health primarily from physiological and biochemical perspectives. Exercise can regulate emotional states through multiple pathways, enhance resistance to depression and anxiety, and promote relaxation and stress relief. These mechanisms include increased release of neurotransmitters such as serotonin, dopamine, and endorphins (Zhang J. et al., 2024); enhanced expression of brain-derived neurotrophic factor (Chen et al., 2021; Gao et al. (2023)) and endogenous cannabinoids (Gao et al. (2023); Li et al., 2025); regulation of the hypothalamic–pituitary–adrenal (HPA) axis and reduction of glucocorticoid receptor sensitivity (Chen et al., 2021); anti-inflammatory and antioxidant effects that reduce neuroinflammation and neural damage (Zhang X. et al., 2024; Zhang et al., 2025); modulation of sympathetic and parasympathetic nervous system balance to improve HRV (Yu et al., 2024); increases in hippocampal volume, activation of the prefrontal cortex, and optimization of the default mode network (Zhang J. et al., 2024); enhancement of myokine release (Zhang et al., 2023); regulation of gut microbiota structure and function (Min et al., 2025); and modulation of interactions between peripheral organs and the central nervous system (Li et al., 2024).

For green and blue landscapes, elemental morphology, spatial layout, and diversity, together with natural sounds such as bird chirping, insect buzzing, wind through leaves, flowing water, and waves, stimulate both visual and auditory systems. Through the thalamus–midbrain pathway, such stimulation can increase dopamine release in the ventral tegmental area, enhance parasympathetic nervous system activity, reduce heart rate and blood pressure, improve HRV, strengthen stress recovery capacity, and alleviate emotional stress (Brewerton et al., 2018; Gianfredi et al., 2021). In addition, fresh air, high concentrations of negative ions, and aromatic vegetation and flowers in green and blue natural environments can activate the olfactory system to regulate the limbic system, enhance *γ*-aminobutyric acid–ergic neuronal activity, and promote cerebral relaxation (Van den Berg et al., 2015; Fang et al., 2023). Green environment exposure can inhibit HPA axis activity, reduce salivary cortisol secretion, and mitigate chronic stress–induced hippocampal damage, whereas blue environment exposure can stimulate oxytocin release, thereby enhancing social connectedness and perceived security (Zajkowska et al., 2022). Furthermore, green and blue environmental exposure can reorganize prefrontal–amygdala functional connectivity, integrate the default mode network, regulate neuroplasticity, reduce negative emotion processing, and alleviate rumination closely associated with depression (Dion-Albert et al., 2022). These environments may also promote mental health by directly or indirectly modulating inflammatory responses (Ruszkiewicz et al., 2019), epigenetic modifications (Wei et al., 2024), and the gut microbiota–gut–brain axis (Agirman and Hsiao, 2021). Collectively, exercise and green and blue environmental exposure can synergistically regulate neuroendocrine responses, immune and inflammatory processes, brain structure and functional remodeling, autonomic nervous system balance, central–peripheral organ interactions, and epigenetic mechanisms, thereby jointly promoting mental health.

Acute Tai Chi exercise can rapidly stimulate the release of neurotransmitters such as dopamine and endorphins (Zhang J. et al., 2024), which synergizes with the immediate physiological relaxation induced by green and blue environmental exposure to accelerate positive emotional changes. This acute synergistic effect does not depend on long-term training accumulation and is therefore particularly suitable as a convenient intervention strategy for immediate emotional regulation in older adults, complementing evidence from studies on cumulative effects of long-term exercise combined with environmental exposure, such as the long-term green exercise research by Han (2017).

### Analysis of the maintenance of the acute effects of subsequent exposure to green and blue environments on short-term emotions of elderly males following the combined effect

The results of this study indicated that when heart rate returned to resting levels after Tai Chi exercise, HRV, blood pressure, and short-term emotional indices in the TJQ, GTJQ, BTJQ, and GBTJQ groups all exhibited significantly positive changes compared with values prior to the experiment. Among these groups, the BTJQ group showed the greatest magnitude of positive emotional change, followed by the GBTJQ, GTJQ, and TJQ groups. These findings suggest that continued exposure to green and blue environments is beneficial for maintaining positive emotional changes in elderly males after the combined intervention. Moreover, the maintenance effects varied according to environment type, with the following order: blue environment exposure > balanced green-blue environment exposure > green environment exposure > conventional environment exposure.

Relatively few studies have examined how sustained exposure to green and blue environments maintains the mental health benefits achieved through combined environmental exposure and exercise interventions. A representative domestic study by Gao et al. (2023) on the positive and negative emotional benefits of green and blue visual exposure combined with exercise found that after a combined intervention of green and blue environment exposure and stationary cycling, indices including SDNN, rMSSD, LF/HF ratio, SBP, DBP, positive emotion scores, and negative emotion scores all remained superior when heart rate returned to resting levels, compared with outcomes under single green or blue exposure or prior to the experiment. These results indicate that sustained green and blue exposure can positively maintain mental health improvements derived from combined environmental exposure and exercise (Gao et al., 2023). An international study reported that 15-min seated viewing of urban street green spaces, ordinary urban green parks, and urban forest parks, combined with 30-min walking interventions, significantly improved perceived restorativeness, subjective vitality, emotions, creativity, and salivary cortisol levels. When participants’ heart rates returned to resting levels, continued exposure to these environments during the recovery period prolonged the maintenance of mental health improvements achieved through the combined interventions, with urban forest parks demonstrating the strongest prolongation effect (Tyrväinen et al., 2014). Another study showed that combined interventions involving simulated natural environment video viewing or sound listening with moderate-intensity stationary cycling significantly improved heart rate, blood pressure, overall emotional disturbance, and perceived stress. Ten minutes after the intervention, no significant differences were observed between immediate post-intervention and 10-min post-intervention emotional states and perceived stress across combined intervention groups, suggesting that continuous exposure to simulated natural environments can better maintain mental health improvements over a longer period (Wooller et al., 2018). During post–Tai Chi recovery in green-blue environments, as physical function is gradually restored, reduced metabolic stimulation from exercise may enhance the brain’s visual, auditory, and olfactory processing of green-blue stimuli, thereby allowing psychological benefits to persist during recovery. The maintenance of acute effects may therefore depend on residual physiological influences of acute environmental exposure and exercise. Continued green and blue environmental stimulation after intervention can further activate the parasympathetic nervous system (Van den Berg et al., 2015), counteract emotional decline during post-exercise physiological recovery, and sustain acute effects for a short duration. These findings provide practical guidance for acute intervention design, indicating that extending environmental exposure time after intervention may optimize the maintenance of acute emotional benefits.

### Limitations and future directions

As an acute intervention study, this research emphasizes the immediate and short-term effects of a single intervention, thereby addressing a gap in studies of acute green and blue environment exposure combined with Tai Chi exercise in elderly males and providing a feasible strategy for scenarios requiring immediate emotional regulation (e.g., community-based psychological counseling for older adults and short-term health interventions). However, the acute effects observed in this study cannot substitute for the cumulative benefits of long-term interventions. Future research could therefore examine the cumulative impact of repeated acute interventions by implementing a series of short-term interventions over time.

It should also be noted that, due to various subjective and objective constraints (e.g., elderly women spending more time on family caregiving responsibilities), female participants were not included in this study. In addition, the study involved only a single low-frequency intervention combining sufficient environmental exposure with exercise and did not incorporate long-term or high-frequency intervention designs. Furthermore, because of limitations associated with outdoor experimental conditions, only two objective indicators (HRV and blood pressure) were measured. Future studies could include additional objective indicators of mental health, such as physiological and biochemical markers (e.g., cortisol, salivary amylase, and brain-derived neurotrophic factor) and neurophysiological indices obtained through non-invasive functional brain imaging techniques (e.g., functional near-infrared spectroscopy). Such approaches would facilitate a more comprehensive exploration of the mechanisms through which green and blue environment exposure combined with exercise improves mental health from psychological, physiological, biochemical, and neurobiological perspectives. Moreover, due to the inherent variability of natural environments and individual differences in emotional baseline levels, this study could not fully meet the criteria of a strictly controlled intervention design. Consequently, the findings are observational and correlational in nature and do not constitute direct evidence for causal inference.

Tai Chi, as a mind–body exercise characterized by autonomous consciousness training, appears particularly suitable for promoting mental health when combined with green and blue environment exposure, especially given the relatively low exercise intensity typically tolerated by older adults. However, whether Tai Chi yields greater positive effects than conventional exercises (e.g., jogging, brisk walking, or cycling) under equivalent exercise volume, intensity, duration, and frequency combined with green and blue environment exposure requires further investigation. The inherent uncontrollability of natural environments also limits the feasibility of strictly randomized controlled trials. With the continued advancement and wider application of virtual reality technologies, more precise randomized controlled trials could be conducted using indoor virtual green and blue environment exposure, enabling accurate control of visual field breadth and depth, refinement of individual difference factors, and inclusion of a broader range of subjective and objective mental health indicators. Such studies could examine both single-session and long-term sufficient exposure combined with exercise, thereby strengthening the evidence base regarding the effects of green and blue environment exposure combined with exercise on mental health.

## Conclusion

Acute exposure to green, blue, and balanced green-blue environments exerted positive effects on short-term emotional health in elderly males, with the magnitude of acute effects ranked as follows: blue environment exposure > balanced green-blue environment exposure > green environment exposure > conventional environment exposure. Building on these acute emotional benefits, subsequent Tai Chi exercise further enhanced short-term emotional health in elderly males. The strength of acute positive effects followed the order: blue environment exposure combined with Tai Chi > balanced green-blue environment combined with Tai Chi > green environment exposure combined with Tai Chi > conventional environment combined with Tai Chi exercise. Following the combined acute effects of environmental exposure and Tai Chi exercise, continued short-term exposure to green and blue environments maintained positive emotional changes during the recovery period. The capacity to maintain acute positive effects was ranked as: continuous blue environment exposure > continuous balanced green-blue environment exposure > continuous green environment exposure > continuous conventional environment exposure.

## Data Availability

The raw data supporting the conclusions of this article will be made available by the authors, without undue reservation.
