# Supplementary material for: An acute intervention experimental study on the effects of green and blue environment exposure combined with tai chi exercise on the emotional health of elderly males
Source: Front Psychol. 2026 Feb 4;17:1743865. doi: 10.3389/fpsyg.2026.1743865 (PMC12913083; doi:10.3389/fpsyg.2026.1743865)
Supplement: Supplementary file 1 [file Table_1.docx]

| Metrics | Group | Descriptive statistics at different time points | | | | Intra-group comparison of differences across different time points（F/ɳ2p/P） | | | | | | Inter-group comparison of differences at the same time point（F/ɳ2p/P） | | | |
| --- | --- | --- | --- | --- | --- | --- | --- | --- | --- | --- | --- | --- | --- | --- | --- |
|  |  | Test1 | Test2 | Test3 | Test4 | Test1-Test2 | Test1-Test3 | Test1-Test4 | Test2-Test3 | Test2-Test4 | Test3-Test4 | Test1 | Test2 | Test3 | Test4 |
| SDNN（ms） | TJQ | 43.84±12.43 | 42.29±13.56 | 40.57±16.80 | 42.51±18.21 | 0.28/0.012/0.598 | 0.42/0.018/0.519 | 0.04/0.002/0.843 | 0.19/0.008/0.664 | 0.01/0.000/0.921 | 0.31/0.013/0.581 | TJQ-GTJQ:0.02/0.001/0.887  TJQ-BTJQ:0.06/0.003/0.809  TJQ-GBTJQ:0.01/0.000/0.921 | TJQ-GTJQ:0.11/0.004/0.738  TJQ-BTJQ:4.01/0.159/0.049  TJQ-GBTJQ:0.72/0.030/0.401 | TJQ-GTJQ:0.15/0.006/0.699  TJQ-BTJQ:3.53/0.278/0.015  TJQ-GBTJQ:2.41/0.096/0.126 | TJQ-GTJQ:0.00/0.000/0.965  TJQ-BTJQ:2.94/0.126/0.028  TJQ-GBTJQ:0.47/0.020/0.496 |
|  | GTJQ | 44.27±11.35 | 41.39±11.64 | 39.58±14.86 | 42.39±12.45 | 0.57/0.024/0.453 | 4.83/0.112/0.048 | 0.15/0.006/0.698 | 0.48/0.021/0.492 | 0.98/0.041/0.326 | 2.27/0.092/0.138 | GTJQ-BTJQ:0.15/0.006/0.698  GTJQ-GBTJQ:0.02/0.001/0.883 | GTJQ-BTJQ:5.12/0.187/0.028  GTJQ-GBTJQ:1.89/0.078/0.174 | GTJQ-BTJQ:6.87/0.212/0.022  GTJQ-GBTJQ:3.94/0.148/0.052 | GTJQ-BTJQ:4.94/0.126/0.044  GTJQ-GBTJQ:0.68/0.028/0.412 |
|  | BTJQ | 42.59±14.52 | 36.41±15.87 | 32.76±17.01 | 37.58±10.91 | 4.98/0.162/0.045 | 8.76/0.284/0.005 | 3.21/0.137/0.042 | 2.15/0.088/0.149 | 0.03/0.001/0.862 | 4.28/0.158/0.044 | BTJQ-GBTJQ:0.08/0.003/0.773 | BTJQ-GBTJQ:0.83/0.034/0.366 | BTJQ-GBTJQ:1.76/0.072/0.191 | BTJQ-GBTJQ:0.31/0.013/0.581 |
|  | GBTJQ | 43.58±13.07 | 39.80±16.28 | 36.69±15.39 | 40.04±13.49 | 0.41/0.018/0.524 | 5.82/0.202/0.007 | 0.89/0.038/0.350 | 1.57/0.065/0.216 | 0.02/0.001/0.887 | 2.91/0.115/0.096 | ------ | ------ | ------ | ------ |
| rMSSD（ms） | TJQ | 28.36±10.23 | 29.44±11.86 | 27.82±9.45 | 29.28±11.54 | 0.23/0.010/0.633 | 0.01/0.000/0.925 | 0.18/0.008/0.673 | 0.29/0.012/0.591 | 0.01/0.000/0.932 | 0.53/0.022/0.470 | TJQ-GTJQ:0.03/0.001/0.862  TJQ-BTJQ:0.14/0.006/0.708  TJQ-GBTJQ:0.00/0.000/0.954 | TJQ-GTJQ:1.59/0.066/0.212  TJQ-BTJQ:4.28/0.161/0.047  TJQ-GBTJQ:6.17/0.178/0.043 | TJQ-GTJQ:3.99/0.136/0.049  TJQ-BTJQ:16.27/0.415/0.001  TJQ-GBTJQ:5.23/0.190/0.026 | TJQ-GTJQ:0.51/0.022/0.478  TJQ-BTJQ:0.89/0.038/0.350  TJQ-GBTJQ:1.49/0.061/0.226 |
|  | GTJQ | 27.82±9.64 | 25.50±8.43 | 22.38±9.46 | 26.72±10.62 | 0.94/0.039/0.336 | 4.17/0.154/0.047 | 0.19/0.008/0.665 | 2.88/0.114/0.097 | 0.02/0.001/0.885 | 2.19/0.090/0.144 | GTJQ-BTJQ:0.28/0.012/0.599  GTJQ-GBTJQ:0.04/0.002/0.843 | GTJQ-BTJQ:4.93/0.129/0.058  TJQ-GBTJQ:0.11/0.004/0.737 | GTJQ-BTJQ:10.74/0.302/0.012  GTJQ-GBTJQ:3.65/0.168/0.056 | GTJQ-BTJQ:0.01/0.000/0.920  GTJQ-GBTJQ:0.23/0.010/0.633 |
|  | BTJQ | 29.39±11.22 | 23.85±12.24 | 18.01±13.67 | 25.18±13.76 | 3.97/0.137/0.046 | 12.83/0.365/0.001 | 1.92/0.079/0.171 | 3.94/0.148/0.052 | 0.67/0.028/0.416 | 1.98/0.081/0.165 | BTJQ-GBTJQ:0.10/0.004/0.751 | BTJQ-GBTJQ:0.01/0.000/0.925 | BTJQ-GBTJQ:2.97/0.117/0.092 | BTJQ-GBTJQ:0.20/0.008/0.656 |
|  | GBTJQ | 28.68±10.52 | 24.61±10.67 | 21.14±10.25 | 25.74±9.08 | 1.42/0.059/0.238 | 8.36/0.275/0.006 | 1.74/0.071/0.194 | 2.53/0.101/0.118 | 0.09/0.004/0.765 | 2.83/0.112/0.100 | ------ | ------ | ------ | ------ |
| LF/HF | TJQ | 2.35±1.97 | 2.29±1.24 | 2.44±1.35 | 2.31±1.03 | 0.03/0.001/0.857 | 0.02/0.001/0.885 | 0.01/0.000/0.912 | 0.01/0.000/0.921 | 0.01/0.000/0.938 | 0.02/0.001/0.893 | TJQ-GTJQ:0.02/0.001/0.885  TJQ-BTJQ:0.02/0.001/0.883  TJQ-GBTJQ:0.01/0.000/0.915 | TJQ-GTJQ:0.54/0.023/0.464  TJQ-BTJQ:1.62/0.067/0.208  TJQ-GBTJQ:0.41/0.018/0.524 | TJQ-GTJQ:0.79/0.033/0.378  TJQ-BTJQ:3.91/0.147/0.053  TJQ-GBTJQ:1.28/0.052/0.262 | TJQ-GTJQ:0.61/0.026/0.439  TJQ-BTJQ:1.83/0.075/0.181  TJQ-GBTJQ:0.77/0.032/0.384 |
|  | GTJQ | 2.26±1.23 | 2.54±1.08 | 2.73±1.45 | 2.59±1.39 | 0.51/0.022/0.478 | 0.87/0.036/0.355 | 0.28/0.012/0.599 | 0.19/0.008/0.664 | 0.01/0.000/0.920 | 0.11/0.005/0.742 | GTJQ-BTJQ:0.05/0.002/0.822  GTJQ-GBTJQ:0.00/0.000/0.959 | GTJQ-BTJQ:2.83/0.112/0.100  GTJQ-GBTJQ:0.01/0.000/0.925 | GTJQ-BTJQ:3.42/0.136/0.063  GTJQ-GBTJQ:0.22/0.009/0.641 | GTJQ-BTJQ:0.01/0.000/0.920  GTJQ-GBTJQ:0.01/0.000/0.930 |
|  | BTJQ | 2.40±1.40 | 2.91±1.44 | 3.32±1.07 | 3.02±1.82 | 0.89/0.038/0.350 | 2.64/0.105/0.110 | 1.24/0.051/0.270 | 0.98/0.041/0.326 | 0.04/0.002/0.843 | 0.53/0.022/0.471 | BTJQ-GBTJQ:0.01/0.000/0.921 | BTJQ-GBTJQ:0.17/0.007/0.681 | BTJQ-GBTJQ:0.47/0.020/0.496 | BTJQ-GBTJQ:0.21/0.009/0.649 |
|  | GBTJQ | 2.28±1.77 | 2.60±1.67 | 2.94±1.36 | 2.67±1.05 | 0.29/0.012/0.591 | 1.53/0.063/0.222 | 0.45/0.019/0.504 | 0.37/0.016/0.545 | 0.01/0.000/0.925 | 0.12/0.005/0.731 | ------ | ------ | ------ | ------ |

Supplementary table 1 Descriptive statistics and statistical test results of HRV indices at different time points among participants in each group

Notes: (1) Intra-group comparisons were performed using repeated measures ANOVA, and inter-group comparisons were performed using repeated measures ANOVA + post-hoc tests; P < 0.05 was considered statistically significant. (2) Test 1: prior to the experiment; Test 2: 20 minutes after environmental landscape viewing; Test 3: immediately after Tai Chi exercise; Test 4:

when heart rate recovered to resting level. (3) TJQ: conventional environment + Tai Chi; GTJQ: green environment + Tai Chi; BTJQ: blue environment + Tai Chi; GBTJQ: balanced green-blue environment + Tai Chi. (4) SDNN: the standard deviation of normal-to-normal R–R intervals; rMSSD: root mean square of successive differences; LF/HF: the ratio of LF to HF.
