# Supplementary material for: An acute intervention experimental study on the effects of green and blue environment exposure combined with tai chi exercise on the emotional health of elderly males
Source: Front Psychol. 2026 Feb 4;17:1743865. doi: 10.3389/fpsyg.2026.1743865 (PMC12913083; doi:10.3389/fpsyg.2026.1743865)
Supplement: Supplementary file 2 [file Table_2.docx]

Supplementary table 2 Descriptive statistics and statistical test results of blood pressure indices at different time points among participants in each group

| Metrics | Group | Descriptive statistics at different time points | | | | Intra-group comparison of differences across different time points（F/ɳ2p/P） | | | | | | Inter-group comparison of differences at the same time point（F/ɳ2p/P） | | | |
| --- | --- | --- | --- | --- | --- | --- | --- | --- | --- | --- | --- | --- | --- | --- | --- |
|  |  | Test1 | Test2 | Test3 | Test4 | Test1-Test2 | Test1-Test3 | Test1-Test4 | Test2-Test3 | Test2-Test4 | Test3-Test4 | Test1 | Test2 | Test3 | Test4 |
| SBP（mmHg） | TJQ | 130.89±10.58 | 131.74±11.82 | 137.42±12.71 | 130.12±10.69 | 0.14/0.006/0.708 | 4.04/0.168/0.049 | 0.02/0.001/0.895 | 6.87/0.233/0.011 | 3.11/0.119/0.083 | 6.95/0.221/0.015 | TJQ-GTJQ:0.03/0.001/0.858  TJQ-BTJQ:0.01/0.000/0.921  TJQ-GBTJQ:0.00/0.000/0.965 | TJQ-GTJQ:1.17/0.048/0.283  TJQ-BTJQ:4.19/0.148/0.051  TJQ-GBTJQ:0.18/0.008/0.673 | TJQ-GTJQ:1.49/0.061/0.226  TJQ-BTJQ:4.47/0.176/0.055  TJQ-GBTJQ:0.47/0.020/0.496 | TJQ-GTJQ:0.21/0.009/0.648  TJQ-BTJQ:2.73/0.109/0.104  TJQ-GBTJQ:0.02/0.001/0.890 |
|  | GTJQ | 129.87±11.47 | 127.45±13.25 | 133.36±10.13 | 128.54±10.48 | 0.49/0.021/0.487 | 1.68/0.069/0.201 | 0.09/0.004/0.765 | 4.23/0.173/0.047 | 0.12/0.005/0.731 | 4.02/0.136/0.037 | GTJQ-BTJQ:0.01/0.000/0.920  GTJQ-GBTJQ:0.05/0.002/0.823 | GTJQ-BTJQ:4.98/0.111/0.057  GTJQ-GBTJQ:1.68/0.069/0.200 | GTJQ-BTJQ:4.82/0.186/0.053  GTJQ-GBTJQ:0.76/0.032/0.386 | GTJQ-BTJQ:3.09/0.118/0.084  GTJQ-GBTJQ:0.34/0.015/0.561 |
|  | BTJQ | 130.57±10.28 | 125.54±11.55 | 127.84±11.27 | 125.60±13.29 | 2.08/0.085/0.046 | 1.93/0.079/0.170 | 2.75/0.092/0.041 | 0.31/0.013/0.580 | 0.00/0.000/0.992 | 1.29/0.053/0.261 | BTJQ-GBTJQ:0.07/0.003/0.792 | BTJQ-GBTJQ:1.14/0.047/0.289 | BTJQ-GBTJQ:0.01/0.000/0.925 | BTJQ-GBTJQ:0.52/0.022/0.474 |
|  | GBTJQ | 131.11±13.05 | 128.71±10.88 | 132.46±13.32 | 129.76±12.04 | 0.42/0.018/0.521 | 0.83/0.034/0.366 | 0.06/0.003/0.810 | 1.89/0.078/0.174 | 0.01/0.000/0.920 | 1.54/0.063/0.221 | ------ | ------ | ------ | ------ |
| DBP（mmHg） | TJQ | 80.37±8.45 | 81.37±9.20 | 78.32±8.46 | 80.76±9.33 | 0.23/0.010/0.633 | 0.45/0.019/0.505 | 0.19/0.008/0.664 | 0.91/0.038/0.343 | 0.00/0.000/0.992 | 1.26/0.052/0.267 | TJQ-GTJQ:0.25/0.011/0.619  TJQ-BTJQ:0.00/0.000/0.965  TJQ-GBTJQ:0.01/0.000/0.920 | TJQ-GTJQ:0.45/0.019/0.505  TJQ-BTJQ:4.00/0.143/0.052  TJQ-GBTJQ:0.89/0.038/0.350 | TJQ-GTJQ:0.15/0.006/0.699  TJQ-BTJQ:0.94/0.039/0.336  TJQ-GBTJQ:3.87/0.124/0.052 | TJQ-GTJQ:0.01/0.000/0.925  TJQ-BTJQ:0.65/0.027/0.423  TJQ-GBTJQ:0.89/0.038/0.350 |
|  | GTJQ | 81.61±9.43 | 79.24±8.12 | 77.43±7.67 | 80.24±7.96 | 0.61/0.026/0.439 | 1.72/0.071/0.196 | 0.34/0.015/0.563 | 0.38/0.016/0.541 | 0.99/0.041/0.325 | 2.83/0.112/0.100 | GTJQ-BTJQ:0.14/0.006/0.709  GTJQ-GBTJQ:0.19/0.008/0.663 | GTJQ-BTJQ:4.29/0.173/0.051  GTJQ-GBTJQ:1.38/0.058/0.245 | GTJQ-BTJQ:1.31/0.054/0.257  GTJQ-GBTJQ:1.97/0.080/0.166 | GTJQ-BTJQ:1.01/0.042/0.319  GTJQ-GBTJQ:0.11/0.004/0.737 |
|  | BTJQ | 80.64±7.98 | 76.12±8.34 | 74.42±8.60 | 77.91±8.73 | 3.13/0.106/0.044 | 5.18/0.185/0.027 | 1.12/0.046/0.295 | 0.89/0.038/0.350 | 1.57/0.065/0.216 | 2.25/0.091/0.140 | BTJQ-GBTJQ:0.20/0.008/0.656 | BTJQ-GBTJQ:0.13/0.005/0.718 | BTJQ-GBTJQ:0.83/0.034/0.366 | BTJQ-GBTJQ:0.15/0.006/0.699 |
|  | GBTJQ | 81.35±8.44 | 78.48±9.01 | 76.54±7.98 | 79.40±8.35 | 1.05/0.044/0.309 | 1.21/0.082/0.049 | 0.39/0.017/0.535 | 0.35/0.015/0.557 | 0.37/0.016/0.546 | 1.52/0.062/0.224 | ------ | ------ | ------ | ------ |

Notes: (1) Intra-group comparisons were performed using repeated measures ANOVA, and inter-group comparisons were performed using repeated measures ANOVA + post-hoc tests; P < 0.05 was considered statistically significant. (2) Test 1: prior to the experiment; Test 2: 20 minutes after environmental landscape viewing; Test 3: immediately after Tai Chi exercise; Test 4: when heart rate recovered to resting level. (3) TJQ: conventional environment + Tai Chi; GTJQ: green environment + Tai Chi; BTJQ: blue environment + Tai Chi; GBTJQ: balanced green-blue environment + Tai Chi. (4) SBP: systolic blood pressure; DBP: diastolic blood pressure.
