# Supplementary material for: An acute intervention experimental study on the effects of green and blue environment exposure combined with tai chi exercise on the emotional health of elderly males
Source: Front Psychol. 2026 Feb 4;17:1743865. doi: 10.3389/fpsyg.2026.1743865 (PMC12913083; doi:10.3389/fpsyg.2026.1743865)
Supplement: Supplementary file 3 [file Table_3.docx]

Supplementary table 3 Descriptive statistics and statistical test results of subjective emotion indices at different time points among participants in each group

| Metrics | Group | Descriptive statistics at different time points | | | | Intra-group comparison of differences across different time points（F/ɳ2p/P） | | | | | | Inter-group comparison of differences at the same time point（F/ɳ2p/P） | | | |
| --- | --- | --- | --- | --- | --- | --- | --- | --- | --- | --- | --- | --- | --- | --- | --- |
|  |  | Test1 | Test2 | Test3 | Test4 | Positive affect（F/η²p/P） | | | | | | Positive affect | | | |
|  |  |  |  |  |  | Test1-Test2 | Test1-Test3 | Test1-Test4 | Test2-Test3 | Test2-Test4 | Test3-Test4 | Test1 | Test2 | Test3 | Test4 |
| Positive affect score | TJQ | 12.56±5.08 | 13.01±4.87 | 13.98±5.29 | 12.61±4.37 | 0.21/0.009/0.648 | 1.25/0.051/0.269 | 0.00/0.000/0.987 | 0.89/0.038/0.350 | 0.84/0.035/0.363 | 1.83/0.075/0.181 | TJQ-GTJQ:0.00/0.000/0.965  TJQ-BTJQ:0.12/0.005/0.729  TJQ-GBTJQ:0.00/0.000/0.987 | TJQ-GTJQ:0.19/0.008/0.663  TJQ-BTJQ:1.57/0.065/0.216  TJQ-GBTJQ:0.42/0.018/0.521 | TJQ-GTJQ:0.02/0.001/0.887  TJQ-BTJQ:3.19/0.121/0.079  TJQ-GBTJQ:0.94/0.039/0.336 | TJQ-GTJQ:0.01/0.000/0.920  TJQ-BTJQ:3.02/0.116/0.087  TJQ-GBTJQ:0.08/0.003/0.774 |
|  | GTJQ | 12.67±6.28 | 13.59±6.42 | 14.11±4.06 | 12.78±6.08 | 0.27/0.011/0.604 | 0.69/0.029/0.411 | 0.01/0.000/0.920 | 0.11/0.004/0.737 | 0.78/0.032/0.381 | 1.57/0.065/0.216 | GTJQ-BTJQ:0.15/0.006/0.698  GTJQ-GBTJQ:0.01/0.000/0.920 | GTJQ-BTJQ:1.98/0.081/0.165  GTJQ-GBTJQ:0.01/0.000/0.925 | GTJQ-BTJQ:3.65/0.136/0.061  GTJQ-GBTJQ:0.15/0.006/0.699 | GTJQ-BTJQ:3.38/0.128/0.072  GTJQ-GBTJQ:0.12/0.005/0.729 |
|  | BTJQ | 13.01±5.43 | 14.49±6.08 | 15.07±5.09 | 14.59±5.34 | 1.52/0.063/0.223 | 3.89/0.146/0.053 | 2.35/0.094/0.131 | 1.11/0.046/0.298 | 0.08/0.003/0.774 | 0.31/0.013/0.580 | BTJQ-GBTJQ:0.16/0.007/0.691 | BTJQ-GBTJQ:0.38/0.016/0.540 | BTJQ-GBTJQ:0.22/0.009/0.641 | BTJQ-GBTJQ:0.51/0.022/0.478 |
|  | GBTJQ | 12.55±6.00 | 13.70±5.66 | 14.50±6.31 | 13.01±5.64 | 0.38/0.016/0.541 | 1.47/0.061/0.229 | 0.04/0.002/0.845 | 0.62/0.026/0.434 | 0.33/0.014/0.568 | 1.98/0.081/0.165 | ------ | ------ | ------ | ------ |
| Metrics | Group | Test1 | Test2 | Test3 | Test4 | Negative affect（Z/P） | | | | |  | Negative affect | | | |
|  |  |  |  |  |  | Test1-Test2 | Test1-Test3 | Test1-Test4 | Test2-Test3 | Test2-Test4 | Test3-Test4 | Test1 | Test2 | Test3 | Test4 |
| Negative affect score | TJQ | 7.15±1.20 | 7.12±0.96 | 7.00±1.04 | 7.09±0.90 | -0.12/0.904 | -0.65/0.515 | -0.28/0.779 | -0.53/0.595 | -0.15/0.880 | 0.37/0.711 | TJQ-GTJQ:2.83/0.112/0.100  TJQ-BTJQ:3.21/0.122/0.079  TJQ-GBTJQ:0.76/0.032/0.386 | TJQ-GTJQ:0.38/0.016/0.541  TJQ-BTJQ:1.89/0.078/0.174  TJQ-GBTJQ:3.65/0.136/0.061 | TJQ-GTJQ:0.22/0.009/0.641  TJQ-BTJQ:3.83/0.331/0.052  TJQ-GBTJQ:3.52/0.253/0.058 | TJQ-GTJQ:0.61/0.026/0.439  TJQ-BTJQ:0.38/0.016/0.540  TJQ-GBTJQ:0.01/0.000/0.920 |
|  | GTJQ | 7.94±0.89 | 7.27±1.07 | 6.78±1.22 | 7.40±0.86 | -1.82/0.069 | -0.93/0.415 | -1.36/0.174 | -0.98/0.327 | -0.72/0.471 | -1.65/0.099 | GTJQ-BTJQ:0.05/0.002/0.822  GTJQ-GBTJQ:1.25/0.051/0.269 | GTJQ-BTJQ:2.83/0.112/0.100  GTJQ-GBTJQ:0.69/0.029/0.411 | GTJQ-BTJQ:3.79/0.319/0.051  GTJQ-GBTJQ:3.41/0.258/0.059 | GTJQ-BTJQ:1.25/0.051/0.269  GTJQ-GBTJQ:0.89/0.038/0.350 |
|  | BTJQ | 8.01±1.20 | 6.64±1.00 | 6.02±0.85 | 6.81±1.34 | -1.97/0.058 | -2.07/0.316 | -1.58/0.114 | -1.93/0.053 | -0.23/0.817 | -2.51/0.012 | BTJQ-GBTJQ:1.89/0.078/0.174 | BTJQ-GBTJQ:0.01/0.000/0.925 | BTJQ-GBTJQ:0.61/0.026/0.439 | BTJQ-GBTJQ:0.51/0.022/0.478 |
|  | GBTJQ | 7.68±0.99 | 6.42±1.11 | 6.10±1.05 | 7.05±0.97 | -2.14/0.051 | -3.01/0.141 | -1.42/0.155 | -1.69/0.091 | -0.11/0.912 | -2.23/0.052 | ------ | ------ | ------ | ------ |

Notes: (1) Intra-group comparisons of positive affect were performed using repeated measures ANOVA (F/η2p/P), and intra-group comparisons of negative affect were performed using nonparametric tests (Z/P) due to non-normal distribution. (2) Inter-group comparisons were performed using repeated measures ANOVA + post-hoc tests (F/η2p/P). (3) P < 0.05 was considered statistically significant. (4) Test 1: prior to the experiment; Test 2: 20 minutes after environmental landscape viewing; Test 3: immediately after Tai Chi exercise; Test 4: when heart rate recovered to resting level. (5) TJQ: conventional nvironment + Tai Chi; GTJQ: green environment + Tai Chi; BTJQ: blue environment + Tai Chi; GBTJQ: balanced green-blue environment + Tai Chi.
